# Synthesis of Gallic Acid-Loaded Chitosan-Grafted-2-Acrylamido-2-Methylpropane Sulfonic Acid Hydrogels for Oral Controlled Drug Delivery: In Vitro Biodegradation, Antioxidant, and Antibacterial Effects

**DOI:** 10.3390/gels8120806

**Published:** 2022-12-08

**Authors:** Chengqun Yu, Xuanbin Chen, Weifeng Zhu, Lijun Li, Mingyan Peng, Yulian Zhong, Abid Naeem, Zhenzhong Zang, Yongmei Guan

**Affiliations:** Key Laboratory of Modern Preparation of Traditional Chinese Medicines, Ministry of Education, Jiangxi University of Chinese Medicine, Nanchang 330004, China

**Keywords:** hydrogel, polyphenol, drug delivery, controlled release, oral formulation, antioxidant

## Abstract

In this study, chitosan (CS) and 2-acrylamido-2-methylpropane sulfonic acid (AMPS)-based hydrogels were formulated by the free radical polymerization technique for the controlled release of gallic acid. Fourier transform infrared spectroscopy (FTIR) confirmed the successful preparation and loading of gallic acid within the hydrogel network. Differential scanning calorimetry (DSC) and thermogravimetric analysis (TGA) confirmed the increased thermal stability of the hydrogels following the crosslinking and polymerization of chitosan and AMPS. In X-ray diffraction analysis (XRD), the crystallinity of the raw materials decreased, indicating strong crosslinking of the reagents and the formation of a new polymeric network of hydrogels. Scanning electron microscopy (SEM) revealed that the hydrogel had a rough, dense, and porous surface, which is consistent with the highly polymerized composition of the hydrogel. After 48 h, the hydrogels exhibited higher swelling at pH 1.2 (swelling ratio of 19.93%) than at pH 7.4 (swelling ratio of 15.65%). The drug release was analyzed using ultraviolet-visible (UV-Vis) spectrophotometer and demonstrated that after 48 h, gallic acid release was maximum at pH 1.2 (85.27%) compared to pH 7.4 (75.19%). The percent porosity (78.36%) and drug loading increased with the increasing concentration of chitosan and AMPS, while a decrease was observed with the increasing concentration of ethylene glycol dimethyl methacrylate (EGDMA). Crosslinking of the hydrogels increased with concentrations of chitosan and EGDMA but decreased with AMPS. In vitro studies demonstrated that the developed hydrogels were biodegradable (8.6% degradation/week) and had antimicrobial (zone of inhibition of 21 and 16 mm against Gram-positive bacteria *Escherichia coli* and *Staphylococcus aureus* as well as 13 mm against Gram-negative bacteria *Pseudomonas aeruginosa*, respectively) and antioxidant (73% DPPH and 70% ABTS) properties. Therefore, the prepared hydrogels could be used as an effective controlled drug delivery system.

## 1. Introduction

Gallic acid (3,4,5-trihydroxybenzoic acid) can be found in a wide variety of fruits and wines, including tea, grapes, and berries. A number of beneficial properties are associated with gallic acid, including antioxidant, anti-diabetic, anti-histaminic, antibacterial, and anti-cancer properties [1,2]. This compound is widely used in food, cosmetics, and pharmaceuticals due to its beneficial properties. However, this drug has a very low bioavailability, which severely restricts its use in clinical settings [3].

Traditional drug delivery system techniques require repeated administration or higher dosages to achieve therapeutic effects, which can result in reduced patient compliance and efficacy, as well as adverse side effects that could be life-threatening. Over the past few decades, a great deal of attention has been focused on issues related to traditional delivery systems and controlled drug delivery systems, such as nanoparticles, liposomes, membranes, and hydrogels [4]. The use of these devices allows drugs to be delivered to cells and tissues over an extended period of time. The potential therapeutic benefits are enhanced efficacy and reduced required dose and, therefore, toxicity [5].

“Hydrogels” consist of three-dimensional structures that are formed either physically or chemically by crosslinking and are capable of retaining more water without compromising their structural integrity [6]. Hydrogels have been extensively studied in the field of biomedical devices and drug delivery because of their biocompatibility and easy control over solute transportation. Hydrogels minimize the disadvantages associated with conventional drug delivery systems, thereby optimizing the therapeutic benefits of the drug [7]. Hydrogels are highly hydrated three-dimensional (3D) crosslinked macromer, homopolymer, or copolymer networks that may be fabricated into a variety of shapes and sizes. The physical and chemical crosslinking of polymer chains causes these molecules to swell when exposed to water while maintaining their structural integrity [8].

The higher water content of hydrogels affects their various properties, including their permeability, surface characteristics, biocompatibility, and mechanical functionality [9]. Hydrogels undergo a significant sol–gel phase transition or volume phase transition as a result of chemical or physical stimuli. Chemical and bio-chemical stimuli include ions, pH, and chemical composition of the solvent, as well as temperature, solvent composition, magnetic and electric fields, and pressure [10]. Various chemical methods can be employed to manufacture hydrogels; they can be prepared through three-dimensional (3D) polymerization whereby hydrophilic monomers are polymerized in the presence of crosslinking agents, or they can be prepared by direct polymerization of hydrophilic polymers [11]. Typically, polymerization is triggered by compounds containing free radicals or by the application of gamma, ultraviolet, or electron beam radiation [12].

Chitosan (CS) has become increasingly popular over the past few years due to its biocompatible, biodegradable, and non-toxic nature, which makes it an excellent material for biomedical use [13]. It is composed of glucosamine and N-acetylglucosamine, which are linked by (1–4) linkages. This polymer is one of the most widely used biomaterials due to its cationic properties, which enable it to bind to negatively charged molecules such as proteins and nucleic acids. The –NH_2_/–OH functional groups of this polymer can be used efficiently for graft polymerization in order to produce hydrogels with favorable physical and chemical properties [14].

Crosslinking with ethylene glycol dimethacrylate (EGDMA) has been widely used in a variety of formulations. The use of EGDMA as a crosslinking agent has been favored owing to its superior release and swelling properties [15]. 2-acrylamido-2-methylpropanesulfonic acid (AMPS) is a hydrophilic monomer containing both ionic and non-ionic groups. This amide monomer displays strong resistance to salts and improved stability in terms of its resistance to hydrolysis owing to its sulfonic functional group. The sulfonic groups in AMPS allow it to be crosslinked with natural polymers to form networks with desired properties [16].

In the current study, we developed chitosan-grafted-2-acrylamido-2-methylpropane sulfonic acid (CS-*g*-AMPS) hydrogels by the free radical polymerization technique for the controlled delivery of gallic acid. This study aims to identify an extended-release formulation that may produce a desired and effective pattern of drug release in order to improve the bioavailability of gallic acid (as gallic acid is rapidly excreted and has a low bioavailability) [1]. Various studies were performed on the prepared hydrogels, including FTIR, XRD, TGA, DSC, SEM, sol–gel analyses, polymer volume fractions, dynamic swelling, drug loading, in vitro drug release, kinetic modeling, percent porosity, biodegradation, and antioxidant and antibacterial analyses. The results of this study indicate that the hydrogels developed can be used as a controlled release system for gallic acid and other similar drugs.

## 2. Results and Discussion

### 2.1. FTIR Analysis

**Figure 1:**
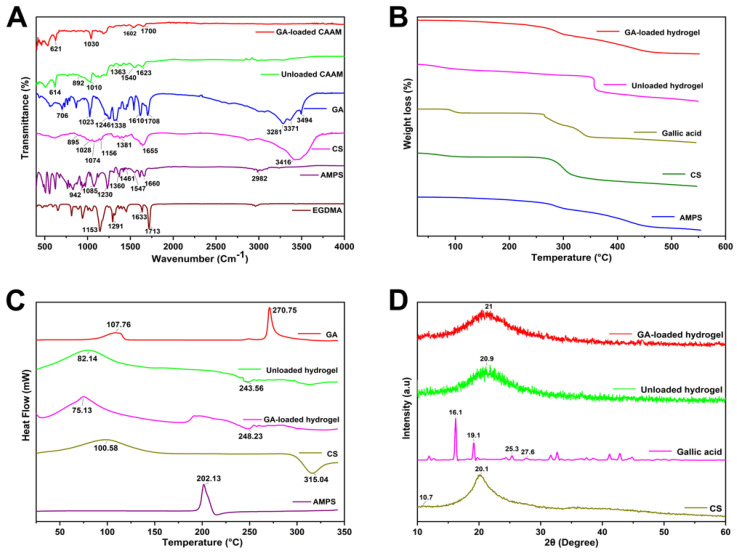
FTIR (**A**), TGA (**B**), DSC (**C**), and XRD (**D**) of CS-*g*-AMPS hydrogels and its components.

FTIR spectra of chitosan, gallic acid, AMPS, EGDMA, and unloaded and drug-loaded hydrogels are shown in Figure 1A. The EGDMA spectrum shows a vibration band at 1713 cm^−1^, associated with stretching vibrations of C=O, and bands at 1633 cm^−1^, 1291 cm^−1^, and 1153 cm^−1^ are associated with stretching vibrations of C=C and C–O, respectively [17]. FTIR spectrum of AMPS shows bands at 1660 cm^−1^ and 1547 cm^−1^, representing C=O bond stretching and N–H bond bending, respectively. The strong bands observed at 1085 cm^−1^ and 942 cm^−1^ correspond to the S–O–C group. The characteristic bands were also present at 2982 cm^−1^ due to –CH stretching of –CH_2_, and at 1230 cm^−1^ due to symmetrical stretching of the S=O functional group, which confirmed the presence of the SO_3_H group in AMPS [18]. The CS absorption band observed at 3416 cm^−1^ was caused by the overlapping of –OH and symmetric N–H stretching vibrations; the vibration band at 1655 cm^−1^ indicates amide I stretching, whereas the bands at 1381, 1156, 1074, and 895 cm^−1^ indicate O–H bending, C–O–C linkage, C–O–C stretching, and N–H out of plane bending, respectively [19]. In addition, a characteristic band at 1028 cm^−1^ indicates C–O stretching vibrations. Characteristic bands of gallic acid were found at 3281 cm^−1^ and 3494 cm^−1^, corresponding to aromatic and carboxylic O–H stretching. Furthermore, the band at 1610 cm^−1^ is associated with ring vibration (C=C) [20]. Moreover, the band at 1708 cm^−1^ is due to C=O stretching in the carboxylic acid. Other researchers have also reported similar results [21]. In the FTIR spectrum of the synthesized unloaded hydrogel, some distinct bands were observed. In addition, slight shifting, overlapping, and disappearance of some of the characteristic bands of pure components occurred within the hydrogels, which are indicators of the formation of new hydrogel structures. Moreover, the bands of CS were shifted from 1028, 1655, and 895 cm^−1^ to 1010, 1623, and 892 cm^−1^, and the bands of AMPS were shifted from 1547 and 1360 cm^−1^ to 1540 and 1363 cm^−1^, respectively.

Similarly, in drug-loaded hydrogels, the bands of GA, such as at 1708, 1610, and 1023 cm^−1^, are shifted to 1700, 1602, and 1030 cm^−1^, respectively. These results suggest that a new polymeric network is formed and the model drug is entrapped within the hydrogel structure [18].

### 2.2. TGA and DSC Analysis

TGA and DSC was performed to assess the thermal behavior of the developed hydrogels (Figure 1B,C). TGA analysis of gallic acid shows initial weight loss due to the loss of loosely attached water molecules. The decomposition of gallic acid was observed to occur in two stages at onset temperatures of 230 °C and 320 °C. The TGA of AMPS showed a weight loss of 6% at 208 °C, but there was a further 20% loss between 210 and 250 °C, indicating dehydration of AMPS. Furthermore, a 20% weight loss was observed within the temperature range of 250–340 °C because of sulfonic acid group degradation [22]. The CS starts to degrade around 50 °C, which is attributed to the loss of moisture from the samples. However, rapid degradation occurs between 260 and 340 °C for all samples, which is attributed to CO_2_ loss [23]. A weight loss of 18% in the developed unloaded hydrogel occurred due to dehydration in the range of 30–345 °C, followed by a 22% weight loss in the range of 340–353 °C, resulting from the polymer bonds breakdown. The polymer network gradually decomposes after 353 °C and continues to degrade until the polymer backbone has been fully degraded. The developed hydrogel degrades slower and at higher temperatures compared to individual reactants due to enhanced strength and interaction between polymer and monomer. The formation of rigid networks and the shifting of endothermic peaks to elevated temperatures result in enhanced stability at elevated temperatures.

GA appears to show an initial peak at 107.76 °C, likely due to the loss of water, and an endothermic peak was observed at 270.75 °C, likely related to its melting point, and was found to be very similar to 267 °C reported in a previous study, confirming its crystalline conformation [24]. DSC thermogram of AMPS shows an endothermic peak at about 202 °C, indicating the decomposition of AMPS [25]. CS shows a melting peak at 98.5 °C in the DSC thermogram, in agreement with the experimental results of Hoang Thai et al. [26]. The unloaded hydrogel exhibited a peak around 82 °C, indicating initial moisture loss, and another peak at 243 °C, which shows the initial degradation. The present study demonstrates that crosslinking alters the thermal properties of the polymer and monomer and indicates that grafts can develop within the polymer as a result of crosslinking [27].

### 2.3. XRD Analysis

Figure 1D depicts the XRD diffractograms of the polymers and hydrogels. Gallic acid shows various notable peaks, i.e., 2θ of 16.1°, 25.3°, and 27.6°, indicating its crystallinity [28]. Chitosan showed a small protuberance at approximately 2θ = 10.7°, and a crystalline peak at 2θ = 20.1°, attributed to hydrogen bonds within and between the crystal planes [29]. The diffractogram of unloaded hydrogel exhibits one broader peak at 2θ = 20.9°, indicating the amorphous nature of the hydrogel. However, diffractogram of the drug-loaded hydrogel shows one broader peak at 2θ = 21° and a small peak at 24.0° as other peaks of the drug disappeared, confirming the presence of gallic acid within the hydrogel.

### 2.4. SEM Analysis

**Figure 2:**
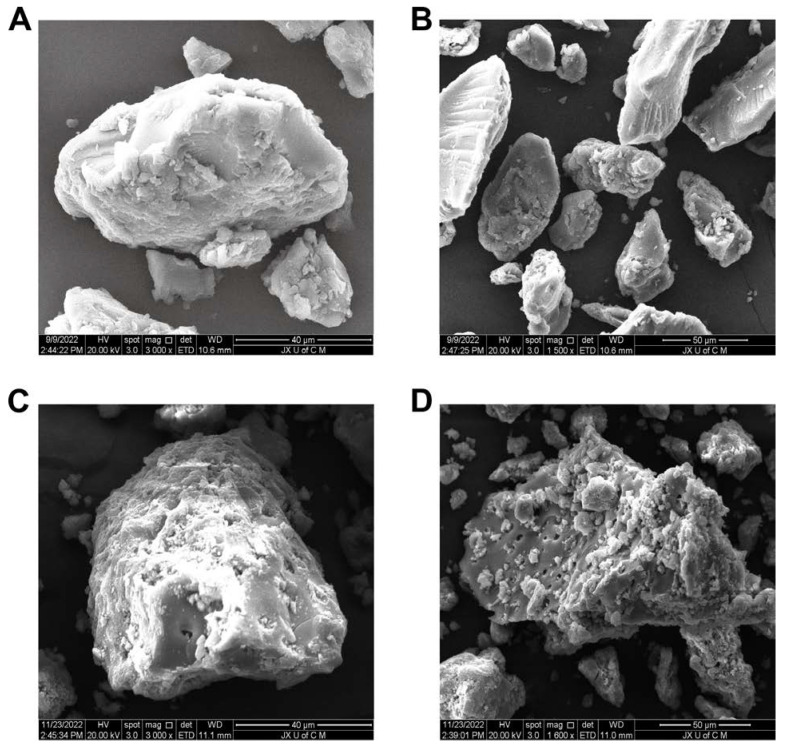
SEM micrographs of unloaded hydrogels at 3000× (**A**) and 1500× (**B**), and drug-loaded hydrogels at 3000× (**C**) and 1600× (**D**).

The surface morphology of the hydrogel system revealed a loose, irregular, porous, dense, and somewhat coarse texture, having both micro- and macropores, showing the porousness and stability of the system (Figure 2). The morphology of the hydrogel shows that polymers have successfully been crosslinked. This porous surface allows water to pass through the interstitial spaces directly, increasing swelling capacity and allowing fluid diffusion within the hydrogel network. It can be seen that the porous and rougher structure of the drug-loaded hydrogels provides a means for transport and release of the adsorbed/encapsulated or attached drug at the intended site. The hydrogel network absorbs water initially through macropores, then gradually through micropores, which increases water absorption [30].

### 2.5. Mechanical Properties Analysis

The mechanical properties of hydrogels are presented in Table 1. Hydrogels used for drug delivery applications need to have certain mechanical characteristics, including tensile strength (TS) and elongation at break (EAB). Tensile strength was increased with an increase in EGDMA content [31]. The mechanical strength of the gel will decrease as the concentration of AMPS increases in the gel because AMPS is an anionic monomer. Several factors may contribute to this phenomenon, including electrostatic attraction and osmotic pressure. Dynamically crosslinked chitosan networks provide excellent fatigue resistance and rapid self-recovery. Other researchers have reported improved mechanical properties and tensile strength of hydrogels. This may be attributed to the increasing density of crosslinks with chitosan content.

### 2.6. Sol–Gel Analysis

**Figure 3:**
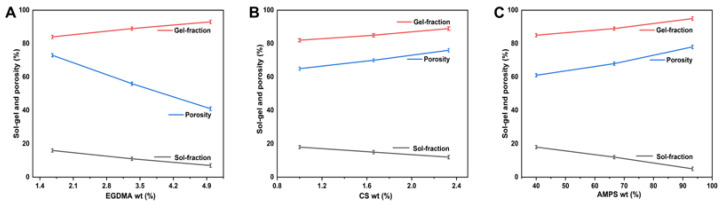
Effect of EGDMA (**A**), CS (**B**), and AMPS (**C**) sol–gel and porosity of CS-*g*-AMPS hydrogel.

Hydrogel formulations were all tested for sol–gel properties (Figure 3A–C). The gel fraction is the crosslinked part of the hydrogel, whereas the sol fraction is the uncrosslinked part. The sol fraction refers to the part of hydrogels that is not crosslinked during the polymerization reaction because there are not enough reactive sites when high amounts of components are used. Sol–gel analyses were conducted on all formulations of hydrogels for the purpose of determining the percentage of crosslinked and uncrosslinked portions. Sol–gel analysis is generally conducted to determine the amounts of polymers that are uncrosslinked. The gel fraction percentage varied from 82.11 to 95.12%, depending on the material ratio. Due to the hydrophilic nature of AMPS, the higher the concentration of AMPS, the more room there is for chemical reactions to take place, increasing the gel fraction. The higher the AMPS content in the total mass, the greater the gel fraction (CAAM-6). EGDMA is a crosslinking agent that can be used to induce the formation of gels [32]. There is a direct relationship between the increase in EDGDMA content and the increase in the gel fraction. The gel fraction of hydrogel increased as chitosan content increased [33].

### 2.7. Porosity Study

The porosity of a hydrogel affects its swelling, loading, and drug release. As pore sizes increase, swelling increases and, consequently, the loading and release of drugs is enhanced. Depending on the reagent ratio, the porosity percentage ranged from 41.25% to 78.36%. Porosity decreases with increasing EGDMA concentration because tight junctions and crosslinks affect hydrogel network flexibility. CAAM-3 has the lowest porosity, which is 41.23%. Porosity is enhanced as AMPS concentrations increase, because stronger electrostatic forces can be generated by the sulfonate groups. A hydrophobic alkyl group can reduce hydrogen bond interactions by forming hydrophobic microregions within AMPS. As a result, the pore and network sizes are larger in the hydrogel preparation, which is consistent with other findings [27]. Specifically, CAAM-6 is the most porous, with a porosity of 78.36%. When the amount of chitosan in the hydrogel is higher, the porosity of the hydrogel is improved. Chitosan improves the surface function of the crosslinker, which improves its porosity and mechanical properties [34]. The porosity of the hydrogel is increased by increasing the concentration of chitosan. Chitosan increases the viscosity of the solution, preventing bubbles from escaping, thereby increasing its porosity.

### 2.8. Biodegradation Analysis

**Figure 4:**
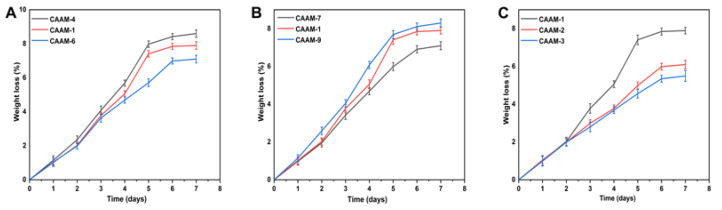
Effect of different weight ratios of hydrogel components on the biodegradation of hydrogels such as AMPS (CAAM-4,1,6) (**A**), CS (CAAM-7,1,9) (**B**), and EGDMA (CAAM-1,2,3) (**C**).

Biodegradation studies were conducted on the constructed hydrogel, as shown in Figure 4A–C. Weight ratio has a significant effect on hydrogel degradation. According to different ratios, hydrogels had a degradability percentage of 5.5% to 8.6%. Studies have shown that with the increase in EGDMA content, the speed of hydrogel degradation slowed down. This may be due to the generation of functional groups that resulted in large quantities of free radicals. Free radicals play an important role in the polymerization reaction, as they strengthen the crosslinked water gel content, leading to a slow rate of degradation. This resulted in the lowest degradation degree for CAAM-3, which was 5.5%. Chitosan is a biodegradable and biocompatible polymer. The weight of alginate/chitosan hydrogels designed by Sibusiso Alven et al. for wound dressings decreased by 80% after two weeks, demonstrating good biodegradability [35]. We found that under the same weight of hydrogel, higher chitosan concentration resulted in a better degradation of the hydrogel.

### 2.9. Swelling Behavior

**Figure 5:**
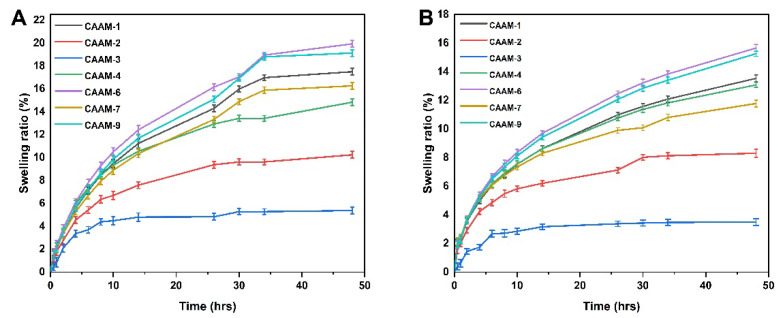
Swelling curves of hydrogels over time at pH 1.2 (**A**) and pH 7.4 (**B**).

Different polymer, monomer, and crosslinker concentrations were used to prepare hydrogels in order to investigate the effects of these concentrations on swelling ratios in different media. In this study, it was found that the swelling of the hydrogel was higher at pH 1.2 than at pH 7.4 (Figure 5). The increased swelling was caused by the ionization of hydroxyl (–OH) functional groups within the hydrogel in water media [36]. Hydrogel swelling percentages ranged from 5.38% to 19.93% at pH 1.2. CAAM-6 had the highest swelling degree at pH 1.2 (19.93%), and CAAM-3 had the lowest swelling degree at pH 1.2 (5.38%). The percent swelling ratio of the hydrogel at pH 7.4 ranged from 3.49% to 15.65%. CAAM-6 had the highest swelling degree at pH 7.4 (15.65%), and CAAM-3 had the lowest swelling degree at pH 7.4 (3.49%). Due to the large number of –CONH_2_ and –SO_3_OH groups in AMPS, increasing its amount will result in a greater degree of equilibrium swelling. Ionization of these groups leads to a greater tendency for these groups to bind to water molecules, which are absorptive, thus, the higher the number of these groups, the greater the absorption [37]. When the concentration of EGDMA is increased, the swelling degree decreases. The porosity of hydrogels decreases as the concentration of EGDMA increases in hydrogels. Therefore, as EGDMA concentration increases, water penetration into the hydrogel network is reduced, resulting in a reduction in swelling [38]. The swelling effect of the hydrogel was increased by increasing the amount of chitosan in the lower pH solution. Similar results were also found by other researchers.

### 2.10. Release and Kinetic Modelling Analysis

**Figure 6:**
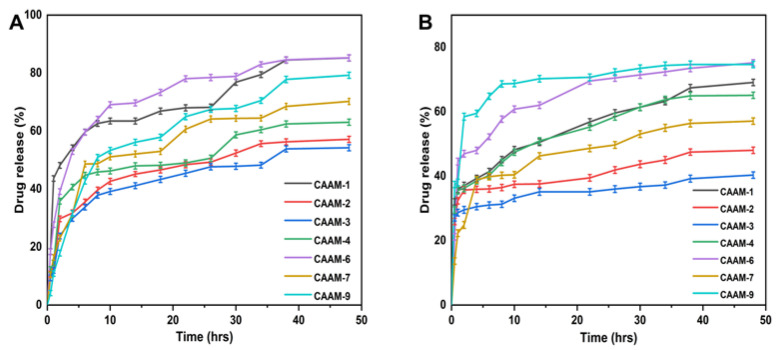
Drug release curves of CS-*g*-AMPS hydrogels at pH 1.2 (**A**) and pH 7.4 (**B**).

The gallic acid release from CS-*g*-AMPS hydrogel was determined at both lower pH (1.2) and higher pH (7.4) values using a UV-Vis spectrophotometer at a wavelength of 220 nm. Drug release from hydrogels is directly related to its swelling in the media [39]. Hydrogel discs absorb water molecules through osmotic pressure gradients when immersed in water. The hydrogel discs swell due to the diffusion of water, creating channels through which the drug is released [40]. Figure 6 shows that the amount of drug released in the buffer at pH 1.2 varies depending on hydrogel composition concentration (54.25% to 85.27%). The drug release rate for CAAM-6 was highest at pH 1.2 (85.27%), whereas the drug release rate for CAAM-3 was lowest at pH 1.2 (54.25%). The drug release rate in the buffer with pH 7.4 was 40.34~75.19%. CAAM-6 showed the highest drug release (75.19%), while CAAM-3 had the lowest drug release (40.34%) at pH 7.4. Gallic acid was released differently at different pH buffers, with its maximum release occurring at pH 1.2. It was found that 85.27% of the drug was released after 48 h.

The regression coefficient value near 1 was considered as the most appropriate model for fitting the drug release data. Table 2 displays regression coefficients (r) for samples containing different concentrations of chitosan (CAAM-1, CAAM-7, CAAN-9). According to their regression coefficient values, these samples followed the Korsmeyer–Peppas model of release kinetics. The regression coefficients for samples (CAAM-1, CAAM-2, CAAM-3) with varying concentrations of the crosslinker EGDMA were also determined. Because the values of the regression coefficients were close to 1, these samples also followed the Korsmeyer–Peppas model. The regression coefficients (r) for the Korsmeyer–Peppas model with varying concentrations of AMPS (CAAM-1, CAAM-4, CAAM-6) confirmed that the release mechanism is controlled by diffusion. Based on the Korsmeyer–Peppas model, the release exponent (*n*) values of all hydrogels (CAAM-1, CAAM-2, CAAM-3, CAAM-4, CAAM-6, CAAM-7, CAAM-9) were between <0.5, indicating a Fickian diffusion process [41].

### 2.11. Structural Parameters of CS-g-AMPS Hydrogels

Several structural characteristics were determined for the synthesized hydrogels, including their average molecular weight between crosslinks, Mc (crosslinking degree), polymer volume fraction, V2,s (the amount of fluid that the network absorbs and retains), solvent interaction degree, χ, crosslinks repeating number, N, and diffusion factor D. The values of a number of structural parameters are presented in Table 3. It is vital to calculate these parameters for hydrogels in order to determine how compatible the solvent is with the polymers used as well as their maximum capacity for uptake and retention of the solvent. Accordingly, the values of V2,s, and χ increased with increasing concentrations of EGDMA, which indicates tighter and stiffer gel structures [42]. It is also evident that Mc and N values decreased as EGDMA concentration increased, because increased crosslinking density was associated with increased amounts of EGDMA, resulting in decreases in Mc and N values. Increasing crosslinking density reduces the swelling properties of the polymeric network, while increasing V2,s increases swelling properties.

### 2.12. Antioxidation Analysis

**Figure 7:**
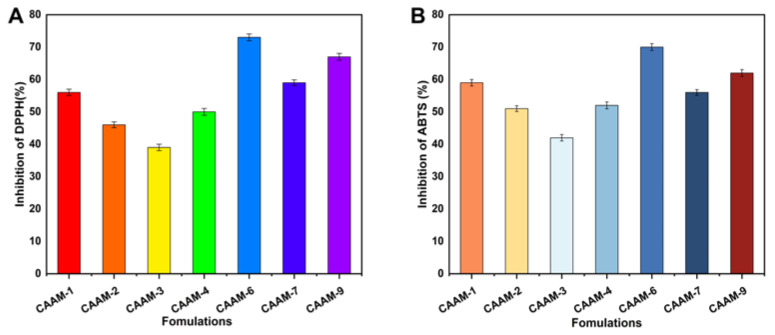
The antioxidant activity of hydrogels using DPPH (**A**) and ABTS (**B**) assay.

Hydrogels were evaluated for their antioxidant activity by scavenging ABTS and DPPH, as shown in Figure 7A,B. Four formulations (CAAM-1, CAAM-6, CAAM-7, and CAAM-9) showed more antioxidant activities. These formulations showed higher swelling and higher release, and the feeding concentrations of chitosan were higher in these formulations. Chitosan polysaccharides have been found to reduce systemic oxidative stress indexes and act as direct antioxidants. Gallic acid has excellent antioxidant properties as well as anti-cancer and antibacterial properties. When gallic acid is loaded into hydrogel, it also exhibits good anti-oxidant properties [43]. Our results show that gallic acid-loaded hydrogels exhibit strong antioxidant properties.

### 2.13. Antibacterial Study

**Figure 8:**
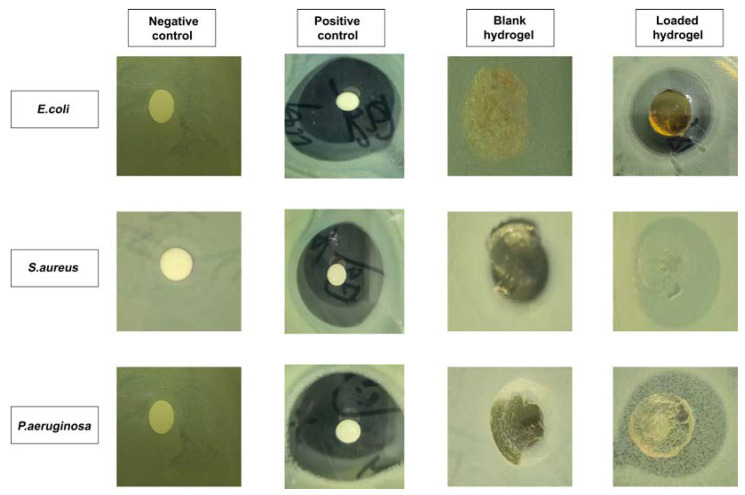
Zones of inhibition of the CS-*g*-AMPS hydrogels against three types of bacteria.

The antibacterial activity of the developed hydrogels was observed against both Gram-positive (*E. coli* and *S. aureus*) and Gram-negative (*P. aeuroginosa*) bacteria. Figure 8 illustrates the zones of inhibition of each type of bacteria. There were no clear inhibition zones in the negative control group or in the blank hydrogel group, while zones of inhibition were evident in the positive control group (27 mm, 29 mm, and 23 mm) and gallic acid-loaded hydrogel (21 mm, 16 mm, and 13 mm) against *E. coli*, *S. aureus*, and *P. aeruginosa*, respectively. The effectiveness of cefepime has been demonstrated against both Gram-positive and Gram-negative bacteria [44]. The antibacterial activity of cefepime was observed against selected strains of bacteria, which exhibited a lower zone of activity against Gram-negative bacteria compared to Gram-positive bacteria. This behavior may be related to the structure of the bacteria’s cell wall. The cell wall of Gram-negative bacteria is composed of three layers: an outer membrane, an inner membrane, and a peptidoglycan wall [45]. Gram-positive bacteria possess thick cell walls, but do not possess an outermost membrane, in contrast to Gram-negative bacteria. This outer membrane serves as a barrier against the external environment for Gram-negative bacteria. As a result, Gram-positive bacteria have a larger inhibitory zone than Gram-negative bacteria.

## 3. Conclusions

The purpose of this study was to fabricate a controlled release hydrogel delivery system for gallic acid using free radical polymerization, by using natural polymers, such as chitosan, with AMPS integrated into its backbone. FTIR, TGA, XRD, and DSC analyses verified that hydrogel networks were developed, as well as that the drug (gallic acid) had been loaded successfully into the hydrogels. SEM analysis revealed that the hydrogels were porous. The swelling ratio of hydrogels showed a pH independent characteristic after 48 h, with a swelling ratio of 19.93% at pH 1.2 and 15.65% at pH 7.4. Additionally, pH-independent drug release characteristics were observed in the developed hydrogels after 48 h at pH 1.2 (85.27%) and pH 7.4 (75.19%), indicating a Fickian diffusion mechanism to release the drug. It was demonstrated that increased polymer ratios and monomer concentrations resulted in prolonged drug release times as well as improved mechanical properties. Furthermore, the hydrogels displayed good porosity (78.36%) and biodegradability (8.6% weight loss after a week). In addition, the developed hydrogels were found to be effective antioxidants when measured against the DPPH assay (inhibition of 73%) as well as the ABTS assay (inhibition of 70%). Additionally, the hydrogels displayed excellent antibacterial activity against *E. coli* (zone of inhibition of 21 mm) and *S. aureus* (zone of inhibition of 16 mm) as well as Gram-negative bacteria *P. aeruginosa* (zone of inhibition of 13 mm). CS-*g*-AMPS hydrogels provide a promising alternative to prolonged delivery of hydrophilic drugs, including gallic acid.

## 4. Materials and Methods

### 4.1. Materials

Chitosan (CS; low molecular weight ≈ 50,000–190,000 Da, with deacetylation degree of ≥75%), 2-acrylamido -2-methyl-1-propanesulfonic acid (AMPS; MW: 207.25 g/moL), ethylene glycol dimethacrylate (EGDMA; MW: 198.22 g/moL), and ammonium persulfate (APS) were obtained from Sigma-Aldrich, Saint Louis, USA. Sodium bisulfite (SHS) was provided by Shanghai Aladdin biochemical technology, Shanghai, China. ABTS (2,2′-azino-bis (3-ethylbenzothiazoline-6-sulfonic acid) and DPPH (2,2- diphenyl-1-picryhydrazyl) were procured from Meilune biological company (China).

Bacteria, such as *Pseudomonas aeruginosa* (*P. aeruginosa*: ATCC27853HBJZ017), *Staphylococcus aureus* (*S. aureus*: ATCC25923HBJZ005), and *Escherichia coli* (*E. coli*: ATCC25922HBJZ087) were provided by Qingdao Hope Biotechnology Co., Ltd., (Qingdao, China).

### 4.2. Synthesis of CS-g-AMPS Hydrogels

**Figure 9:**
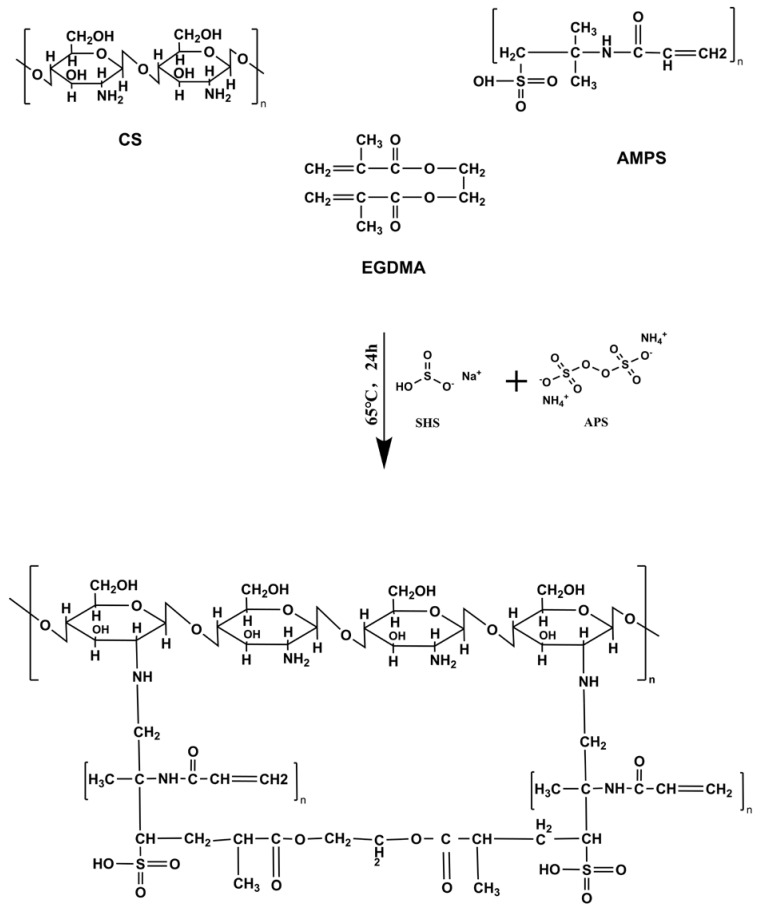
Proposed chemical structure of CS-*g*-AMPS hydrogels.

Hydrogels were formulated in different batches using a free radical polymerization method that involved grafting monomers onto polymer networks [46]. Chitosan (natural polymer) solutions were prepared with (1% w/v) aqueous acetic acid. APS and SHS were used as a combination of initiator and co-initiator of the reaction, respectively. The SHS was mixed with distilled water while stirring, and the APS was added drop-by-drop to the initiator mixture and stirred gently. In a similar manner, the clear aqueous solution of AMPS was prepared at room temperature by stirring continuously. The initiator/co-initiator mixture was incorporated drop-by-drop into the monomer solution while stirring constantly to obtain a clear solution. A mixture of AMPS and initiator/co-initiator was poured drop-wise into the chitosan solution, which was stirred continuously, followed by the addition of EDGMA crosslinker to the chitosan solution. In the final step, sufficient water was added to the reaction mixture and stirred well. Then, the mixture was placed in an ultrasonic bath for a few minutes while nitrogen bubbles were used to remove air. Afterwards, aluminum foil was applied to the molds to cover the clarified solution. After that, the samples were placed in a preheated water bath at 50 °C for 1 h, and the temperature was gradually increased and maintained at 65 °C overnight. After 24 h, clear, transparent hydrogels were formed. Hydrogels formed in the glass molds were removed from the water bath and cooled at room temperature. The hydrogel formed in the molds was removed and cut into discs measuring 8 mm in diameter. After washing the discs with ethanol and water (50:50), they were transferred to individually labeled Petri dishes. Discs were dried at 40 °C for 1 week to achieve a constant weight. Table 4 provides a series of CS-*g*-AMPS-based hydrogel formulations with varying levels of polymer, monomer, and crosslinker concentrations. Figure 9 illustrates a schematic illustration of CS-*g*-AMPS-based hydrogels.

### 4.3. Drug Loading

A swelling-diffusion technique was used to incorporate gallic acid into the hydrogels [47]. Briefly, gallic acid was dissolved in a pH 7.4 buffer, and the dried hydrogel was submerged in the solution for three days and gently stirred. Hydrogel discs were removed from the solution after three days, dried completely, and weighed, and the amount of drug was calculated by subtracting the weight of the loaded hydrogel from the weight of the unloaded hydrogel. Furthermore, the amount of drug loaded in the hydrogel was measured using UV-spectrometry at a wavelength of 220 nanometers after extracting the drug with a buffer (pH 7.4).

(1) $$\begin{array}{c} \text{Drug loading}=\mathrm{Drug}\;\mathrm{loaded}\;\mathrm{hydrogel}-\mathrm{Unloaded}\;\mathrm{hydrogel} \end{array}$$

### 4.4. In Vitro Characterization

#### 4.4.1. Drug and Hydrogel Compatibility Study

The drug-formulation interaction was studied using FTIR spectroscopy with attenuated total reflectance (ATR) technique performed using a Spectrum Two FTIR spectrometer (Perkin Elmer, Buckinghamshire, UK). A series of scans were conducted between the scanning ranges of 400 and 4000 cm^−1^ to determine the spectra of gallic acid-loaded and unloaded hydrogel formulations, as well as their purified components.

#### 4.4.2. Thermal Stability Study

The thermal stability of synthesized hydrogels as well as their purified components was assessed with an Exstar TG/DTA6300TG thermogravimetric analyzer (SII Nano, Tokyo, Japan) and differential scanning calorimetry (Perkin Elmer, Buckinghamshire, UK). The thermogravimetric analyzer was employed to assess weight change as a function of temperature. The weight profile was calibrated using reference standards. Chitosan, AMPS, and the synthesized hydrogel formulations were placed in aluminum pans (0.5 to 5 mg each). The percentage of weight loss was determined by increasing the temperature at a rate of 10 °C/min while flowing inert nitrogen at a rate of 10 mL/min. Differential scanning calorimetry (DSC) was employed to evaluate the melting points of AMPS, Chitosan, and the synthesized hydrogel formulation. Sapphire standards were used to calibrate calorimeters for heat capacity. Indium was used as a standard for determining the cell constant and temperature.

#### 4.4.3. Determination of Crystallinity

The crystallinity of hydrogel and other raw materials was measured by X-ray diffraction (TD-3500, Shenzhen, China) at 30 kV and 20 mA with irradiation of target (CuKα) at 30 kV and 20 mA [48]. A number of scans were performed at a speed of 2 degrees/minute at a 2-theta of 10 to 60°. Jade/MDI software was used to process the data. The crystallinity of a material is determined by its peaks. Amorphous materials are characterized by diffuse peaks, whereas pure materials have sharp peaks. A wide range of samples were tested, including AMPS, Chitosan, and unloaded and gallic acid-loaded hydrogels.

#### 4.4.4. Morphological Analysis

A scanning electron microscope (Quanta 250, FEI, Brno-Královo Pole, Czech Republic) was employed to observe hydrogel shape, microstructure, and porosity. The surface morphology of vacuum-dried samples was investigated in a cross-sectional view under an accelerated current of 15 kV after the samples were mounted on an aluminum stub, coated with gold by sputtering, and observed under an accelerated current of 20 kV.

#### 4.4.5. Determination of the Mechanical Properties

Each formulation was tested for mechanical properties such as tensile strength (*TS*) and elongation at break (*EAB*) using a TA.XT plus texture analyzer (Stable Micro Systems, Godalming, UK) equipped with a stainless-steel spherical diameter probe at a test speed of 1.0 mm/s. *TS* and *EAB* are calculated based on the force and displacement applied to the hydrogel by the probe [49].

(2) $$\begin{array}{c} TS=\frac{Fm}{Th} \end{array}$$

(3) $$\begin{array}{c} EAB=\frac{\sqrt{D^{2}+R^{2}}}{R}-1 \end{array}$$

where *Fm* represents the force of the probe applied to the hydrogel and *Th* represents its thickness. *R* is the radius of the plate and *D* is the distance between the probe and the hydrogel from the initial point of contact to the breakage point.

#### 4.4.6. Determination of Sol–Gel Fraction

The fabricated hydrogels were subjected to sol–gel analyses to determine the proportion of soluble, uncrosslinked, and insoluble crosslinked segments. The gel fraction of a hydrogel is insoluble while the sol fraction is the soluble fraction [50]. The sol–gel analysis was conducted using the Soxhlet extraction method. Hydrogel discs were measured and placed in flasks with distilled water at specific volumes. Round bottom flasks were connected to condensers. They were then left at 85 °C for 12 h in order to complete the extraction process. Once the hydrogel disc was extracted, it was dehydrated completely in a vacuum oven. After dehydrating, it was weighed again. Following are the equations that were used to determine the sol–gel fraction of the hydrogels:

(4) $$\begin{array}{c} \mathrm{Sol}\;\mathrm{fraction}\;\%=\frac{R1-R2}{R2\;}\times 100 \end{array}$$

(5) $$\begin{array}{c} \mathrm{Gel}\;\mathrm{fraction}=100-\mathrm{Sol}\;\mathrm{fraction} \end{array}$$

where R1 refers to the weight of the hydrogel before extraction and R2 corresponds to the weight of the hydrogel after extraction and drying.

#### 4.4.7. Porosity Study

The porosity of the synthesized hydrogel was evaluated using the solvent replacement technique. Briefly, dried hydrogel discs (A1) were immersed for 4 days in absolute ethanol (purity > 99.9%). Afterwards, the hydrogel discs were removed, blotted with filter paper, and then weighed again (A2). Disc thickness and diameter were also measured. Porosity was calculated using the following equation [51]:

(6) $$\begin{array}{c} \mathrm{Porosity}\;\mathrm{percentage}\;\left(\%\right)=\frac{A2-\;A1\;}{\rho v\;}\times 100 \end{array}$$

where ρ is the density of absolute ethanol, A1 is the dry hydrogel weight, A2 is the weight of the disc after removal from ethanol, and V represents the volume of the hydrogel following swelling.

#### 4.4.8. Biodegradation Study

The biodegradation study was conducted on CS-*g*-AMPS hydrogels in a pH 7.4 phosphate buffer at 37 + 0.5 °C. Thus, in order to determine the degradation of the hydrogels at the same intervals, i.e., 1, 2, 3, 4, 5, 6, and 7 days, the hydrogels were immersed in a pH 7.4 buffer solution for one week [47]. The hydrogel discs were removed within the indicated time frame, dehydrated completely at 40 °C in a vacuum oven, weighed again, and placed in a buffer solution of pH 7.4. The hydrogels were observed for a week to assess degradation. Hydrogel degradation can be determined using the following equation:

(7) $$\begin{array}{c} D=\frac{L1-L2}{L1} \end{array}$$

where D represents degradation, *L*1 represents the weight of the dry sample, and *L*2 represents the weight of the sample after immersion at time (t).

### 4.5. CS-g-AMPS Hydrogel Network Parameters

There are several parameters commonly used to evaluate the structure and characteristics of hydrogels in the swollen state, including the volume fraction of the swollen polymer (V2,s), crosslinking molecular weight (Mc), the parameters related to the interaction between the polymer and the solvent (χ), and the number of crosslinks that connect them (N) [47].

#### 4.5.1. Diffusion Coefficient (D)

The rate of diffusion of a substance depends on the nature and segmental mobility of the polymer network. Diffusion coefficient was estimated using the following formula:

(8) $$\begin{array}{c} D=\pi {\left(\frac{h\times \theta }{4\times qeq}\right)}^{2} \end{array}$$

where q*eq* represents equilibrium swelling, θ indicates the slope of the linear part of the swelling curve, and h represents the thickness of the hydrogel disc.

#### 4.5.2. Polymer Volume Fraction (V2,s)

Polymer volume fraction is represented by V2,s and indicates the proportion of polymer in its fully swollen state. It is calculated using the following formula:

(9) $$\begin{array}{c} V2,s=\frac{1}{\mathrm{Veq}\;} \end{array}$$

#### 4.5.3. Average Molecular Weight between Crosslinks (Mc)

The following equation can be used to calculate the average molecular weight between crosslinks:

(10) $$\begin{array}{c} \mathrm{Mc}=\frac{\mathrm{dpVs}\left({V\;}^{\frac{1}{3}}2,s-V2,\frac{s}{2}\right)}{\ln\left(1-V2,s\right)+V2,s+{\chi V}^{2}2,s} \end{array}$$

where dp is the polymer density, Vs is the solvent molar volume and χ is the Flory–Huggins parameter that describes the interaction of polymers and solvents.

#### 4.5.4. Solvent Interaction Parameters (χ)

Solvent interaction parameters can be determined using the Flory–Huggins theory.

(11) $$\begin{array}{c} Χ=\frac{\ln\left(1-V2,s\right)+V2,s}{V^{2}2,s} \end{array}$$

where V2,s represents polymer volume fraction in equilibrium swelling state.

#### 4.5.5. Number of Crosslinks between Repeating Units (N)

N is calculated by utilizing the data of Mc. The following equation was used for the calculation:

(12) $$\begin{array}{c} N=\frac{2\mathrm{Mc}}{\;\mathrm{Mr}\;} \end{array}$$

Mr represents the repeating unit’s molar mass. The equation below can be used to calculate it:

(13) $$\begin{array}{c} \mathrm{Mr}=\frac{\mathrm{mCSMCS}\;+\;\mathrm{mAMPSMAMPS}\;+\;\mathrm{mEGDMAMEGDMA}}{\;\mathrm{mCS}\;+\;\mathrm{mAMPS}\;+\;\mathrm{mEGDMA}} \end{array}$$

where m represents the mass and M indicates the molar mass of CS, AMPS, and EGDMA, respectively.

### 4.6. Equilibrium Swelling Ratio (ESR)

The equilibrium-swelling ratio (*ESR*) was calculated using phosphate buffer (pH 1.2 and 7.4). Briefly, dry hydrogels were weighed and placed in the buffer, and their weight was measured at predefined times. The weights of hydrogels were recorded until equilibrium was reached. Percentage swelling was calculated as follows:

(14) $$\begin{array}{c} ESR=\frac{\mathrm{Nt}-\mathrm{Ni}}{\mathrm{Ni}}\times 100 \end{array}$$

where Nt is the weight of hydrogel at time t and N*_i_* is the initial weight of the hydrogel. 

### 4.7. In Vitro Drug Release and Kinetics Modeling

Drug release from the synthesized hydrogels was tested at two pH levels, namely pH 1.2 and pH 7.4, using a UV-spectrometer. The hydrogel discs containing the drug were submerged in 900 mL of phosphate buffer solutions of pH 1.2 and pH 7.4 at 37 °C, at 50 rpm. Fresh medium of the same volume was introduced at regular intervals after aliquots of 5 mL were obtained. Filtered samples were analyzed in triplicate with a UV-Vis spectrophotometer (T6 New Century; Beijing GM) at 220 nm wavelength.

(15) $$\begin{array}{c} \mathrm{Drug}\;\mathrm{release}\%=\frac{\left(\mathrm{Amount}\;\mathrm{of}\;\mathrm{released}\;\mathrm{drug}\right)}{\left(\mathrm{Amount}\;\mathrm{of}\;\mathrm{loaded}\;\mathrm{drug}\;\right)}\times 100 \end{array}$$

Several parameters can influence the release of drugs from hydrogel matrices, including polymer chain relaxation and swellability, matrix type, drug type, and the pH of the releasing solution, among others. A controlled release hydrogel expands due to solvent diffusion, thereby ensuring controlled release. Models such as Zero-order, First-order, Higuchi, and Korsmeyer–Peppas were used to determine the pattern of drug release.

(16) $$\begin{array}{c} \mathrm{Zero}-\mathrm{order}\;\mathrm{kinetics}\;\;\;\mathrm{Ft}=K0t \end{array}$$

where K0 refers to the apparent rate constant associated with zero-order drug release and Ft refers to the amount released at time t.

(17) $$\begin{array}{c} \mathrm{First}\;\mathrm{order}\;\mathrm{kinetics}\;\;\;\ln\left(1-F\right)=-K1t \end{array}$$

where K1 is the first order constant related to the rate at which drugs are released at time t and F represents the amount of drug released at time t.

(18) $$\begin{array}{c} \mathrm{Higuchi}\;\mathrm{model}\;F=K2t^{\frac{1}{2}} \end{array}$$

where F represents the amount of drug released in time t and K2 represents the Higuchi constant. It is based on two hypotheses: (I) solubility is lower than matrix drug quantity, (II) drug diffusion is one-way.

(19) $$\begin{array}{c} \mathrm{Korsmeyer}-\mathrm{Peppas}\;\mathrm{model}\;\frac{\mathrm{Mt}}{M\infty }=K3t^{n} \end{array}$$

where Mt represents the amount of water absorbed at time t and M∞ specifies the amount of water absorbed at equilibrium. K3 reflects the geometrical and structural characteristics of the gels, and n is the exponent of the release. If n is 0.45, then it represents Fickian release, but if n is more than 0.45 and less than 1 it represents non-Fickian release.

### 4.8. Antioxidant Activities

#### 4.8.1. DPPH Assay

A DPPH free radical scavenging assay was performed to determine the antioxidant activity of the hydrogels. About 20 mg of each sample was soaked in methanol and left for 24 h in the dark at room temperature. The sample solution was then mixed with 1 mL of DPPH methanol solution (0.1 mM). Afterwards, the mixture was vigorously shaken and then placed in a dark place to incubate for 30 min. The DPPH scavenging activity was calculated by measuring the absorption of the solution at 517 nm with a UV-Vis spectrophotometer. It was calculated using the following equation [52].

(20) $$\begin{array}{c} \mathrm{DPPH}\left(\%\right)=\frac{A0-\;A}{A0}\times 100 \end{array}$$

where A0 and A represent control and sample absorbances.

#### 4.8.2. ABTS Assay

The ABTS assay was conducted to assess the radical-scavenging activity of gallic acid-loaded hydrogels. A 1:1 ratio of 7.4 mM ABTS to 2.4 mM potassium persulfate solution was used to induce ABTS radicalization. The hydrogels were then mixed with the ABTS solution and incubated for 30 min at 37 °C. The absorbance was measured at 730 nm using a spectrometer. The ABTS scavenging effect was calculated using the following formula [53]:

(21) $$\begin{array}{c} \mathrm{ABTS}\;\mathrm{scavenging}\;\mathrm{effect}\;\left(\%\right)=\frac{A0-A1}{A0}\times 100 \end{array}$$

A0 denotes the ABTS absorbance, whereas A1 indicate the hydrogels absorbance.

### 4.9. Antibacterial Activity

Nutrient agar was prepared by dissolving agar media in deionized water and autoclaving it at 121 °C/15 psi atmospheric pressure for 30 min. The media was poured into Petri dishes and allowed to cool at room temperature in order to solidify it. The strains of *Pseudomonas aeruginosa*, *Staphylococcus aureus*, and *Escherichia coli* were swabbed and cultured for 24 h. The plates were divided into four categories: unloaded hydrogel (control), gallic acid-loaded hydrogel, positive control (1 mg/mL Cefepime solution), and negative control. The zone of inhibition was determined after incubating the plates for 24 h at 37 °C [54].

### 4.10. Statistical Analysis

The numerical data were expressed as mean ± SD. The statistical difference between adjacent data was determined using a two-way ANOVA followed by a Tukey’s post-hoc test. *p*-values were calculated to determine whether there is a significant difference between swelling and drug release profiles, represented by * *p* < 0.05, ** *p* < 0.01, and *** *p* < 0.001.

## Figures and Tables

**Table 1:** Mechanical properties and drug loading in hydrogels with different material ratios (CAAM-1 to CAAM-9). CAAM represent the hydrogel formulation code.

Formulation Codes	Thickness (mm)	TS (N/mm)	EAB (%)	Drug Loaded per 1 g Hydrogel (g)
CAAM-1	0.99	0.439	43.9	0.479
CAAM-2	1.05	0.796	61.5	0.339
CAAM-3	1.24	0.842	69.3	0.310
CAAM-4	1.19	0.421	41.0	0.412
CAAM-5	0.99	0.439	43.9	0.479
CAAM-6	1.36	0.958	72.5	0.514
CAAM-7	1.59	0.443	51.3	0.396
CAAM-8	0.99	0.439	43.9	0.479
CAAM-9	0.98	0.802	66.2	0.456

**Table 2:** Release kinetics of gallic acid from CS-*g*-AMPS hydrogels.

F. Codes	pH	Zero Order		First Order		Higuchi Model		Korsmeyer–Peppas Model	
		K_o_ (h^−1^)	r^2^	K_1_ (h^−1^)	r^2^	K_2_ (h^−1^)	r^2^	r^2^	*n*
CAAM-1	1.2	0.594	0.9368	0.010	0.9603	3.461	0.9958	0.9969	0.444
	7.4	0.591	0.9578	0.007	0.9488	3.514	0.9919	0.9980	0.390
CAAM-2	1.2	0.387	0.8795	0.004	0.8902	2.310	0.9730	0.9872	0.324
	7.4	0.314	0.8631	0.003	0.8716	1.898	0.9649	0.9888	0.270
CAAM-3	1.2	0.199	0.7639	0.002	0.7703	1.229	0.9009	0.9461	0.240
	7.4	0.137	0.7773	0.001	0.7817	0.849	0.9112	0.9475	0.220
CAAM-4	1.2	0.424	0.8959	0.005	0.9067	2.504	0.9804	0.9909	0.353
	7.4	0.341	0.8969	0.004	0.9044	2.034	0.9793	0.9928	0.305
CAAM-5	1.2	0.594	0.9368	0.010	0.9603	3.461	0.9958	0.9969	0.444
	7.4	0.591	0.9578	0.007	0.9488	3.514	0.9919	0.9980	0.390
CAAM-6	1.2	0.811	0.9377	0.010	0.9551	4.685	0.9945	0.9959	0.432
	7.4	0.667	0.9380	0.008	0.9514	3.888	0.9961	0.9980	0.386
CAAM-7	1.2	0.721	0.9306	0.009	0.9465	4.195	0.9928	0.9953	0.405
	7.4	0.507	0.9196	0.006	0.9309	2.987	0.9906	0.9967	0.346
CAAM-8	1.2	0.594	0.9368	0.010	0.9603	3.461	0.9958	0.9969	0.444
	7.4	0.591	0.9578	0.007	0.9488	3.514	0.9919	0.9980	0.390
CAAM-9	1.2	0.625	0.9313	0.007	0.9449	3.630	0.9928	0.9951	0.413
	7.4	0.502	0.9291	0.006	0.9396	2.943	0.9937	0.9976	0.360

**Table 3:** Flory–Huggins network parameters of CS-*g*-AMPS hydrogels.

F. Codes	V_2,s_	χ	M_c_	M_r_	N	D × 10^−5^ (cm^2^ s^−1^)
CAAM-1	0.032	0.510	3013.6	238.10	25.313	0.022
CAAM-2	0.076	0.526	2860.0	237.21	24.113	0.015
CAAM-3	0.157	0.559	1364.1	236.33	11.544	0.049
CAAM-4	0.067	0.523	1082.3	257.32	8.412	0.052
CAAM-5	0.032	0.510	3013.6	238.10	25.313	0.022
CAAM-6	0.033	0.511	3289.4	229.53	28.662	0.061
CAAM-7	0.037	0.512	1247.3	203.82	12.239	0.055
CAAM-8	0.032	0.510	3013.6	238.10	25.313	0.022
CAAM-9	0.043	0.514	5333.3	250.33	42.610	0.048

**Table 4:** Feed composition of different formulations of CS-*g*-AMPS hydrogels per 100 g.

Formulation Codes	Chitosan (g)	AMPS (g)	APS/SHS (g)	EGDMA (g)
CAAM-1	0.5	20	0.3/0.3	**0.5**
CAAM-2	0.5	20	0.3/0.3	**1**
CAAM-3	0.5	20	0.3/0.3	**1.5**
CAAM-4	0.5	**12**	0.3/0.3	0.5
CAAM-5	0.5	**20**	0.3/0.3	0.5
CAAM-6	0.5	**28**	0.3/0.3	0.5
CAAM-7	**0.3**	20	0.3/0.3	0.5
CAAM-8	**0.5**	20	0.3/0.3	0.5
CAAM-9	**0.7**	20	0.3/0.3	0.5

Note: Bold letters represent higher feeding amounts.

## Data Availability

Not applicable.
